# The prognostic significance of the circumferential resection margin in esophageal squamous cell carcinoma patients without neoadjuvant treatment

**DOI:** 10.1186/s12885-022-10276-1

**Published:** 2022-11-16

**Authors:** Zhaoyang Yang, Hua Lin, Zhen Wang, Lulu Rong, Xuchen Zhang, Lin Wang, Jianjun Qin, Xuemin Xue, Yin Li, Liyan Xue

**Affiliations:** 1 Department of Pathology, National Cancer Center/National Clinical Research Center for Cancer/Cancer Hospital, Chinese Academy of Medical Sciences, and Peking Union Medical College, Beijing, 100021 China; 2 Department of Medical Record, National Cancer Center/National Clinical Research Center for Cancer/Cancer Hospital, Chinese Academy of Medical Sciences, and Peking Union Medical College, Beijing, 100021 China; 3 Department of Thoracic Surgery, National Cancer Center/National Clinical Research Center for Cancer/Cancer Hospital, Chinese Academy of Medical Sciences, and Peking Union Medical College, Beijing, 100021 China; 4 Beijing Chaoyang Hospital, Capital Medical University, Beijing, 100021 China; 5 Department of Pathology, Yale University School of Medicine, 20 York Street, East Pavilion 2-608 C New Haven, New Haven, CT 06510 USA; 6 Beijing Friendship Hospital, Capital Medical University, Beijing, 100021 China

**Keywords:** Esophagus, Squamous cell carcinoma, Circumferential resection margin, Prognosis

## Abstract

**Background:**

Circumferential resection margin (CRM) is very important in esophageal cancer, but its diagnostic criteria has not been unified. The College of American Pathologists (CAP) and the Royal College of Pathologists (RCP) provide two different criteria. The aim of this study is to evaluate the long-term prognostic significance of CRM status with different CRM criteria in esophageal squamous cell carcinoma (ESCC).

**Methods:**

Influence of CRM status according to the CAP and RCP criteria on long-term survival of 838 patients with resected pT3 tumors and without neoadjuvant therapy was analyzed. Patients stratified into three groups on the basis of tumor distance from the CRM (CRM > 1 mm, 0-1 mm, and 0 mm) were also analysed.

**Results:**

Positive CRM was found in 59 (7%) patients according to the CAP criteria and 317 (37.8%) patients according to the RCP criteria. Univariate and multivariate survival analysis showed that CRM status, according to three different criteria, was independent prognostic factor. However, subgroup analysis showed that the prognostic value of CRM status was limited to certain metastatic lymph node load. In pN0 subgroup, patients with CRM > 1 mm had better prognosis than patients with CRM 0-1 mm. Patients with CRM 0 mm had worse outcome than patients with CRM > 0 mm in pN1-2 subgroup. But CRM status had no prognosis value in pN3 subgroup.

**Conclusions:**

The CRM status is an important prognostic factor in ESCC patients, but this effect was limited to patients without or with less lymph node metastasis (pN0-2). In clinical practice, we recommend the 1 mm-three-tier criteria as it provides more prognostic value than the traditional two-tier criteria.

## Background

Esophageal squamous cell carcinoma (ESCC) is one of the most common malignancies worldwide, especially in East Asia, such as China. In recent decades, the application of new technologies, devices, and neoadjuvant therapy has led to great progress in the diagnosis and treatment of esophageal cancer. Whether neoadjuvant therapy was undergone or not, surgery is still the cornerstone of the treatment of locally advanced esophageal cancer. As is well known, complete resection is the most important principle of surgical resection of the primary tumor. It has been reported that positive proximal and distal resection margins have significant association with worse prognosis in terms of recurrence and survival [1–3]. Even though the role of positive CRM in esophageal cancer has been investigated for decades, but it is still controversial. Many articles showed that there was significant relationship between positive CRM and local–regional recurrence, disease-free survival (DFS), and overall survival (OS) [4–9]. However, some studies were unable to show an effect of positive CRM on OS and tumor recurrence [10–12].

The College of American Pathologists (CAP) criteria and the Royal College of Pathologists (RCP) criteria are the two most commonly used criteria for the definition of positive or negative CRM. Tumor found at the resection margin is defined as positive CRM according to the CAP criteria [13], whereas tumor found at or within 1 mm of the resection margin is defined as positive CRM according to the RCP criteria [14]. The difference between the CAP and RCP criteria is whether CRM 0-1 mm should be interpreted as positive or not. If carcinoma is identified microscopically within 1 mm of the CRM after esophagectomy, physicians often face difficulties in selecting proper therapeutic options because little is known about the subspecialized cutoff points between 0 and 1 mm for positive CRM. To fulfill clinical needs and resolve disputes, some studies also proposed other CRM criteria. Yang et al. [4] suggested CRM 600 μm as the optimal cut-off point, and this modified CRM criteria had better prognostic power than the traditional criteria in ESCC patients. In addition to the above two-tier criteria, other studies also proposed the 1 mm-three-tier criteria (CRM > 1 mm, 0-1 mm, and 0 mm) [8] and the 500 μm-three-tier criteria (CRM > 500 μm, 0-500 μm, and 0 μm) [6]. However, most of the previous studies included patients with different pathologic T status and histologic subtypes, and various preoperative therapies (with or without neoadjuvant therapy), that could not represent the true significance of CRM status. As T1 or T2 tumor with CRM involvement is considered as surgical failure, and surgery is a rare treatment option in T4 tumor, so they should be separated from T3 tumor. Moreover, esophageal squamous cell carcinoma and adenocarcinoma are completely different disease entities [15–17], so they should be studied separately. In addition, neoadjuvant therapy can affect the prognosis [18, 19], which may affect the true value of a positive CRM on outcomes. The purpose of this study is to appraise the long-term prognostic significance of CRM status with different CRM criteria in ESCC and select the appropriate diagnostic criteria.

## Methods

### Patients

From March 1999 to July 2007, pT3 ESCC patients who underwent esophagectomy without prior neoadjuvant therapy at National Cancer Center/National Clinical Research Center for Cancer/Cancer Hospital, Chinese Academy of Medical Sciences, and Peking Union Medical College were consecutively analyzed. Exclusion criteria included incomplete resection (defined as cases with the presence of microscopic tumor within 1 mm of the proximal or distal resection margins), distant metastasis, postoperative mortality (within 30 days or in-hospital mortality), and patients with other malignancies that occurred before or after the diagnosis of primary esophageal cancer. In addition, specific sub-types of ESCC, including basaloid squamous cell carcinoma, spindle cell squamous cell carcinoma and verrucous squamous cell carcinoma were also excluded.

### Surgical procedure

The most common procedures for tumors located in the middle-lower esophagus were modified Sweet through left thoracotomy or modified Ivor-Lewis through right thoracotomy approach with intrathoracic anastomosis. For tumors located in the upper esophagus, modified McKeown with cervical anastomosis were performed. All procedures involved two-field lymph node dissection.

### Pathologic examination

**Fig. 1:**
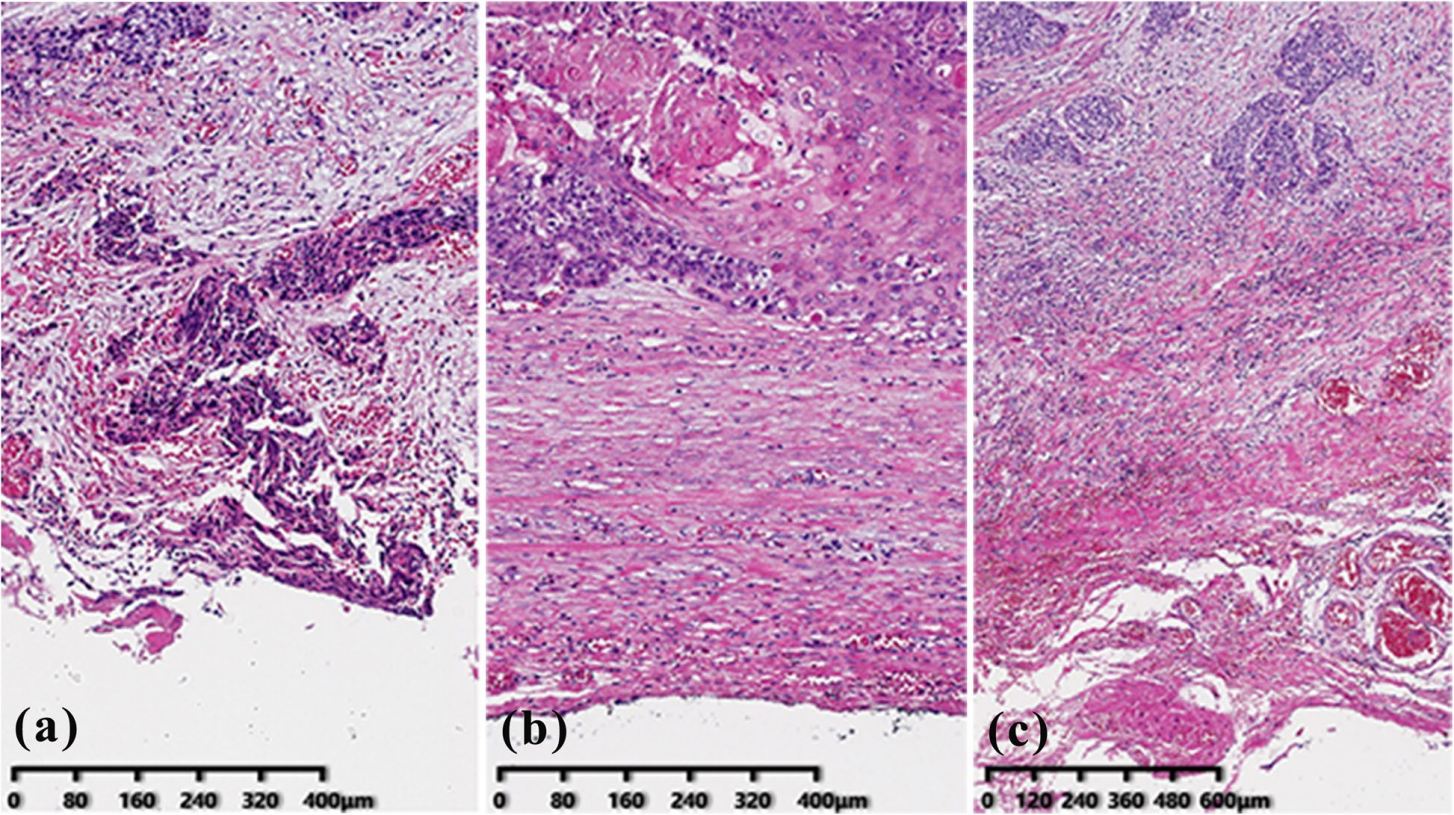
Photomicrographs of resected sections of esophageal cancer. **a** Tumor cells at the circumferential margin (CRM 0 mm), R1 according to CAP and RCP criteria. (Original magnification, × 100). **b** tumor cells within 1 mm of circumferential margin (CRM 0-1 mm), R0 according to CAP criteria, and R1 according to RCP criteria. (Original magnification, × 100). **c** tumor cells within more than 1 mm circumferential margin (CRM > 1 mm), R0 according to CAP and RCP criteria. (Original magnification, × 40)

All original slides from the enrolled patients were reviewed by two certified pathologists. The CRM distance was measured from the deepest tumor cells to the vertical margin. The CRM status was identified by the CAP criteria (R0 and R1), the RCP criteria (R0 and R1), and the 1 mm-three-tier CRM criteria (CRM > 1 mm, 0-1 mm, and 0 mm), respectively (Fig. 1). In addition, all specimens were reviewed for lymph node status, extranodal invasion of lymph node metastasis, the degree of differentiation, lymphovascular invasion (LVI), and perineural invasion (PNI).

### Follow-up

Follow-up data were mainly gathered from clinical notes. Most patients were followed up every 3 months for the first two years after operation, every 6 months until the fifth year, and then annually for 10 years. For those patients who did not come for a follow-up visit, data were gathered by phone calls, and/or mail contact with patients or their next of kin. Survival was measured in months; cancer-related death was scored as an event; the death of any other causes was scored as the end of follow-up. OS time was recorded as the number of months from the date of surgery to the date when the death occurred or to the time of the last follow-up, at which point, the data were censored. DFS time was recorded as the number of months from the date of surgery to the date when the tumor recurred or death due to disease progression.

### Statistical analysis

Patient’s age was analyzed after categorization. Pearson’s chi-square test was used for categorical variables. Kaplan–Meier method was used to plot the survival curves, and the log-rank analysis was used to evaluate differences in prognosis between groups. Prognostic factors for OS and DFS were calculated by using univariate and multivariate Cox regression analyses. Multivariate survival analysis was performed on factors that achieved statistical significance (*P* < 0.05) on univariate analysis. All statistical analyses were performed using the SPSS software package (version 25.0; IBM Corp, Armonk, NY, USA). A *P* value of less than 0.05 was considered statistically significant.

## Results

A total of 939 patients with pT3 ESCC were included, but follow-up was completed in 838 patients (89.2%). Of these 838 patients, the median follow-up was 35 months (range 2–120 months). The male to female ratio was 4.1:1 (672:166) with the median age of 60 years at surgery (range 31–82 years). CRM positive (R1) was found in 59 (7%) patients according to the CAP criteria and 317 (37.8%) patients according to the RCP criteria. LVI, PNI, and lymph node metastasis were found in 446 (53.2%), 521 (62.2%), and 406 (48.4%) patients, respectively. Extranodal invasion occurred in 226 (52.2%) of the 406 patients with lymph node metastases. A total of 225 patients (26.8%) received post-operative adjuvant therapy, and 172 of these patients (76.4%) had lymph node metastasis. The clinicopathologic parameters of the entire cohort are summarized in Table 1.

**Table 1:** Summary of clinical and histopathological characteristics of the 838 pT3N0-3M0 esophageal squamous cell carcinoma patients, the 5-year (5-yr), 10-year (10-yr) overall survival (OS) rate (%) and the 3-year (3-yr), 5-year (5-yr) disease-free survival (DFS) rate (%)

Characteristic		N (%)	5-yr OS (%)	10-yr OS (%)	*P* value	3-yr DFS (%)	5-yr DFS (%)	*P* value
Sex	Male	672(80.2)	45.7	39.9	0.881	46.2	40.9	0.704
	Female	166(19.8)	44.5	33.3		49.4	37.5	
Age (years)	≤ 60	464(55.4)	48.9	44.2	0.007	50.3	44.0	0.029
	> 60	374(44.6)	41.2	31.7		42.5	35.3	
Tumor location	Upper thoracic	119(14.2)	47.6	39.6	0.694	49.6	41.9	0.893
	Middle thoracic	486(58.0)	45.9	41.7		46.8	40.2	
	Lower thoracic	233(27.8)	43.7	33.3		45.6	39.2	
Degree of differentiation	Well	376(44.9)	49.1	41.8	0.001	53.1	44.9	< 0.001
	Moderate	392(46.8)	44.8	37.6		43.5	37.9	
	Poor	70(8.4)	29.9	27.8		32.6	27.9	
LVI	No	392(46.8)	55.9	48.5	< 0.001	57.7	50.9	< 0.001
	Yes	446(53.2)	36.4	29.7		37.5	30.9	
PNI	No	317(37.8)	54.3	46.2	< 0.001	55.5	48.0	0.001
	Yes	521(62.2)	40.1	34.0		41.5	35.3	
pN	N0	405(48.3)	61.6	52.6	< 0.001	63.8	56.1	< 0.001
	N1	238(28.4)	40.3	34.2		41.1	34.6	
	N2	140(16.7)	21.1	16.9		22.6	16.9	
	N3	55(6.6)	13.7	13.7		9.0	6.8	
CRM CAP criteria	R0	779(93.0)	47.2	39.8	< 0.001	48.5	41.6	< 0.001
	R1	59(7.0)	22.9	22.9		26.0	20.8	
CRM RCP criteria	R0	521(62.2)	50.6	44.2	< 0.001	51.4	44.8	0.001
	R1	317(37.8)	37.0	29.3		39.3	32.5	
CRM three-tier criteria	> 1 mm	521 (62.2)	50.6	44.2	< 0.001	51.4	44.8	< 0.001
	0-1 mm	258(30.8)	40.3	30.6		42.4	35.2	
	0 mm	59 (7.0)	22.9	22.9		26.0	20.8	
**Adjuvant therapy**	No	613(73.2)	50.1	41.4	< 0.001	44.9	38.4	< 0.001
	Yes	225(26.8)	33.2	27.5		27.0	24.8	
Extranodal invasion	No	207(47.8)	37.8	30.8	< 0.001	38.5	32.3	< 0.001
	Yes	226(52.2)	24.3	21.7		24.6	19.2	

*CAP* College of American Pathologists, *CRM* Circumferential resection margin, *LVI* Lymphovascular invasion, *RCP* Royal College of Pathologists, *PNI* Perineural invasion

### Overall Survival and CRM status

**Fig. 2:**
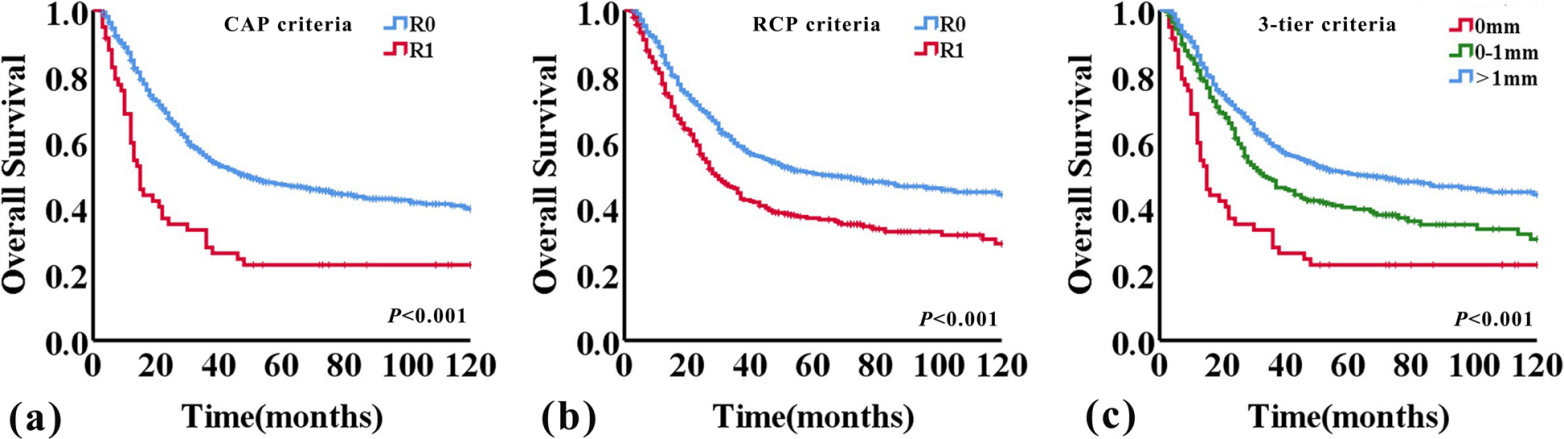
Kaplan–Meier curves for overall survival according to CRM status

The median OS time for the entire study population was 44 months (95%CI 34.2–53.8 months). The 5- and 10-year OS rates were 45.5% and 38.6%, respectively. The median OS of patients who were diagnosed as R0 and R1 according to CAP criteria were 49 months (95%CI 35.7–62.3 months) and 15 months (95%CI 11.7–18.3 months), respectively (*P* < 0.001). Median OS of patients who were diagnosed as R0 and R1 according to the RCP criteria were 66 months (95%CI 39.3–92.7 months) and 29 months (95%CI 23.0–35.0 months), respectively (*P* < 0.001). The Kaplan–Meier survival curve is presented in Fig. 2. Patients with R1 had a significantly shorter OS than those with R0, according to either RCP or CAP criteria used (*P* < 0.001, both; log-rank test). When applying the 1 mm-three-tier stratification system for CRM status, the median OS of patients with CRM 0 mm, 0-1 mm, and > 1 mm were 15 months (95%CI 11.7–18.3 months), 33 months (95%CI 24.3–41.7 months), and 66 months (95%CI 39.3–92.7 months), respectively (*P* < 0.001). And there was significant difference between groups with CRM 0 mm versus CRM 0-1 mm, CRM 0 mm versus CRM > 1 mm, and CRM 0-1 mm versus CRM > 1 mm (*P* = 0.002, *P* < 0.001, and *P* = 0.001, respectively, log-rank test) (Fig. 2c).

The OS of older patients was significantly worse compared with that of young patients. Similarly, cases with poor differentiation, LVI, PNI, higher number of lymph node metastasis, extranodal invasion, and received adjuvant therapy had worse outcome, and the relevant survival data are shown in Table 1.

Univariate Cox proportional hazards model indicated a significant relationship between OS and CRM status (according to either the CAP, or RCP, or 1 mm-three-tier criteria), the patient’s age, degree of tumor differentiation, LVI, PNI, pN, extranodal invasion, and adjuvant therapy (Table 2). Multivariate Cox regression analysis was performed with risk factors that were statistically significant on univariate analysis, except for extranodal invasion. Extranodal invasion is confined to the patients with lymph node metastasis, so it is not suitable for inclusion in multivariate survival analysis. The results of multivariate Cox proportional hazards analysis suggested that the patient’s age, tumor differentiation, pN and CRM status (according to the CAP and RCP criteria) were independent prognostic factors for OS (Table 2). But the difference between CRM 0-1 mm and CRM > 1 mm was not statistically significant (*P* = 0.100).

**Table 2:** Univariate and Multivariate Cox proportional hazards analysis of various prognostic factors and their relationship to overall survival

		Univariate		Multivariate (CAP)		Multivariate (RCP)		Multivariate (three-tier criteria)	
Parameter		HR (95% CI)	*P* value	HR (95% CI)	*P* value	HR (95% CI)	*P* value	HR (95% CI)	*P* value
Sex	Male/Female	0.983(0.783–1.233)	0.882						
Age (years)	≤ 60/ > 60	1.285(1.069–1.545)	0.007	1.017(1.006–1.028)	0.002	1.018(1.007–1.029)	0.001	1.018(1.007–1.029)	0.001
Tumor location	Upper thoracic	1							
	Middle thoracic	0.995(0.756–1.312)	0.974						
	Lower thoracic	1.086(0.806–1.464)	0.586						
Degree of differentiation	Well and Moderate/Poor	1.656(1.231–2.228)	0.001	1.353(1.000–1.832)	0.050	1.400(1.037–1.890)	0.028	1.357(1.003–1.836)	0.048
**LVI**	No/Yes	1.724(1.426–2.084)	< 0.001	1.200(0.977–1.473)	0.082	1.206(0.983–1.479)	0.073	1.189(0.969–1.460)	0.098
PNI	No/Yes	1.500(1.233–1.825)	< 0.001	1.226(1.001–1.502)	0.049	1.212(0.989–1.485)	0.064	1.211(0.988–1.484)	0.065
pN	N0	1		1		1		1	
	N1	1.783(1.420–2.237)	< 0.001	1.697(1.338–2.151)	< 0.001	1.625(1.282–2.060)	< 0.001	1.673(1.319–2.123)	< 0.001
	N2	3.219(2.519–4.114)	< 0.001	2.820(2.160–3.681)	< 0.001	2.721(2.084–3.553)	< 0.001	2.759(2.112–3.605)	< 0.001
	N3	4.536(3.248–6.334)	< 0.001	3.727(2.615–5.311)	< 0.001	3.756(2.632–5.361)	< 0.001	3.694(2.590–5.268)	< 0.001
CRM CAP criteria	R0/R1	2.107(1.543–2.877)	< 0.001	1.814(1.321–2.492)	< 0.001				
CRM RCP criteria	R0/R1	1.517(1.260–1.826)	< 0.001			1.295(1.072–1.564)	0.007		
CRM three-tier criteria	> 1 mm	1						1	
	0-1 mm	1.378(1.128–1.683)	0.002					1.185(0.968–1.452)	0.100
	0 mm	2.350(1.706–3.238)	< 0.001					1.933(1.394–2.680)	< 0.001
**Adjuvant therapy**	No/Yes	1.467(1.205–1.785)	< 0.001	1.072(0.869–1.323)	0.516	1.079(0.875–1.331)	0.478	1.073(0.870–1.324)	0.511
**Extranodal invasion**^**a**^	No/Yes	1.518(1.206–1.912)	< 0.001						

*CAP* College of American Pathologists, *CRM* Circumferential resection margin, *LVI* Lymphovascular invasion, *RCP* Royal College of Pathologists, *PNI* Perineural invasion

^a^ This factor related to prognosis were not examined in the multivariate analysis

### Disease-free survival

**Fig. 3:**
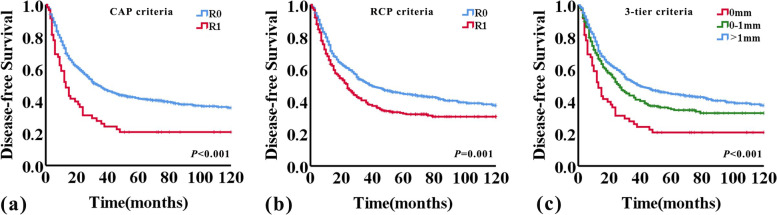
Kaplan–Meier curves for disease-free survival according to CRM status

The median DFS time for the entire study population was 31 months (95%CI 26–36 months). The 3- and 5-year DFS rates were 46.9% and 40.2%, respectively. When using the CAP criteria, the median DFS was 34 months (95%CI 27.8–40.2 months) and 13 months (95%CI 8.9–17.1 months) for patients with R0 and R1 (*P* < 0.001), respectively, while using the RCP criteria, the median DFS was 39 months (95%CI 26.3–51.7 months) for R0 and 24 months (95%CI 19.2–28.8 months) for R1 (*P* = 0.001). The DFS of patients diagnosed as R1 was significantly shorter compared with that of patients diagnosed as R0, according to either CAP or RCP criteria used (*P* < 0.001 and *P* = 0.001, respectively; log-rank test) (Fig. 3a and b). When applying the 1 mm-three-tier criteria for CRM status, the median DFS for patients with CRM 0 mm, CRM 0-1 mm, and CRM > 1 mm was 13 months (95%CI 8.9–17.1 months), 27 months (95%CI 21.4–32.6 months), and 39 months (95%CI 26.3–51.7 months), respectively (*P* < 0.001). And there was significant difference between groups with CRM 0 mm versus CRM 0-1 mm, CRM 0 mm versus CRM > 1 mm, and CRM 0-1 mm versus CRM > 1 mm (*P* = 0.008, *P* < 0.001, and *P* = 0.018, respectively, log-rank test) (Fig. 3c).

The DFS time of older patients was significantly shorter compared with that of young patients. Similarly, cases with poor differentiation, LVI, PNI, higher number of lymph node metastasis, extranodal invasion, and received adjuvant therapy had worse outcome, and the relevant survival data are shown in Table 1.

Univariate Cox proportional hazards model identified a significant relationship between DFS and CRM status (according to either the CAP, or RCP, or 1 mm-three-tier criteria), the patient’s age, degree of tumor differentiation, LVI, PNI, pN, extranodal invasion, and adjuvant therapy (Table 3). Multivariate analyses of the above-mentioned prognostic factors confirmed R1 using the CAP criteria as an independent predictor for DFS. Patient’s age, LVI, pN also remained an independent prognostic factor (Table 3). CRM status, according to the RCP criteria, failed to be an independent prognostic factor. Although, CRM status, according to the three-tier criteria, was an independent prognostic factor, but the difference between groups CRM 0-1 mm versus CRM > 1 mm was not statistically significant (*P* = 0.664).

**Table 3:** Univariate and Multivariate Cox proportional hazards analysis of various prognostic factors and their relationship to disease-free survival

		Univariate		Multivariate (CAP)		Multivariate (RCP)		Multivariate (three-tier criteria)	
Parameter		HR (95% CI)	*P* value	HR (95% CI)	*P* value	HR (95% CI)	*P* value	HR (95% CI)	*P* value
Sex	Male/Female	0.960(0.775–1.188)	0.707						
Age (years)	≤ 60/ > 60	1.213(1.018–1.446)	0.031	1.015(1.005–1.026)	0.003	1.015(1.005–1.026)	0.003	1.015(1.005–1.026)	0.003
Tumor location	Upper thoracic	1							
	Middle thoracic	1.031(0.793–1.341)	0.818						
	Lower thoracic	1.068(0.801–1.423)	0.655						
Degree of differentiation	Well and Moderate/ Poor	1.556(1.161–2.085)	0.003	1.276(0.947–1.718)	0.109	1.306(0.971–1.757)	0.078	1.275(0.946–1.717)	0.110
**LVI**	No/Yes	1.733(1.447–2.077)	< 0.001	1.248(1.025–1.518)	0.027	1.261(1.037–1.534)	0.020	1.245(1.022–1.515)	0.029
PNI	No/Yes	1.363(1.132–1.640)	0.001	1.079(0.890–1.308)	0.438	1.072(0.884–1.299)	0.482	1.076(0.887–1.305)	0.458
pN	N0	1		1		1		1	
	N1	1.813(1.462–2.247)	< 0.001	1.667(1.331–2.087)	< 0.001	1.615(1.290–2.023)	< 0.001	1.660(1.324–2.080)	< 0.001
	N2	3.021(2.389–3.821)	< 0.001	2.581(1.998–3.335)	< 0.001	2.531(1.957–3.272)	< 0.001	2.565(1.983–3.319)	< 0.001
	N3	4.882(3.529–6.753)	< 0.001	3.900(2.760–5.512)	< 0.001	3.852(2.721–5.452)	< 0.001	3.881(2.744–5.489)	< 0.001
CRM CAP criteria	R0/R1	1.803(1.331–2.443)	< 0.001	1.575(1.157–2.145)	0.004				
CRM RCP criteria	R0/R1	1.362(1.140–1.627)	0.001			1.130(0.942–1.355)	0.189		
CRM three-tier criteria	> 1 mm	1						1	
	0-1 mm	1.255(1.036–1.521)	0.020					1.044(0.859–1.270)	0.664
	0 mm	1.945(1.424–2.655)	< 0.001					1.601(1.166–2.199)	0.004
Adjuvant therapy	No/Yes	1.617(1.340–1.952)	< 0.001	1.216(0.996–1.485)	0.055	1.231(1.009–1.503)	0.041	1.216(0.996–1.485)	0.055
Extranodal invasion^a^	No/Yes	1.556(1.247–1.943)	< 0.001						

*CAP* College of American Pathologists, *CRM* Circumferential resection margin, *LVI* Lymphovascular invasion, *RCP* Royal College of Pathologists, *PNI* Perineural invasion

^a^ This factor related to prognosis were not examined in the multivariate analysis

### CRM status, lymph node status, OS, and DFS

**Fig. 4:**
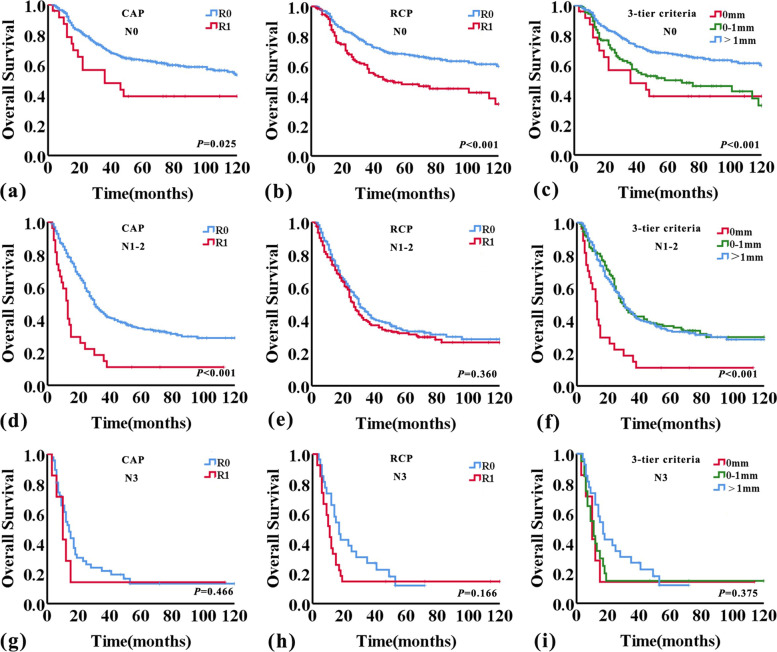
Kaplan–Meier curves showing overall survival according to CRM status assessed by RCP, CAP, and 1 mm-three-tier criteria adjusted for lymph node status

**Fig. 5:**
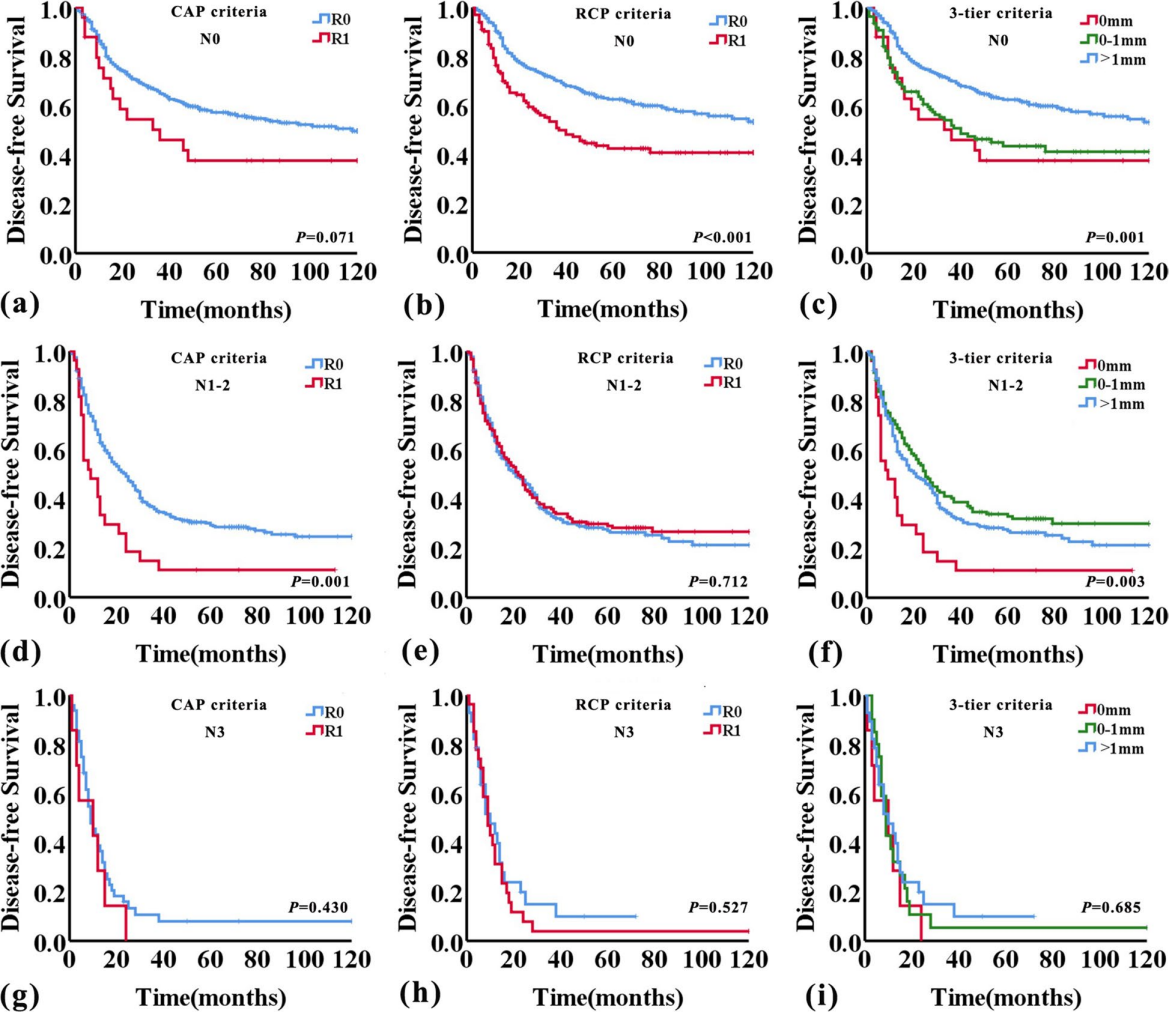
Kaplan–Meier curves showing disease-free survival according to CRM status assessed by RCP, CAP, and 1 mm-three-tier criteria adjusted for lymph node status

As previously described, pN and extranodal invasion were both important prognostic factors, and whether they would affect the predictive value of CRM status was uncertain. Then we analyzed the correlation of CRM status with pN and extranodal invasion of lymph nodes metastasis (Table 4). The result showed that CRM status had a significant correlation with pN (*P* = 0.005), but not extranodal invasion of lymph nodes metastasis (*P* = 0.056). Overall survival and disease-free survival curves were then analysed further for pN0, pN1-2, and pN3 subgroups using the CAP and RCP criteria, as well as the 1 mm-three-tier criteria. This analysis showed good separation of the OS and DFS curves within the pN0 and pN1-2 groups applying either the CAP or RCP, or the 1 mm-three-tier criteria, but not the pN3 group (Figs. 4 and 5). Within the pN0 group, patients with CRM > 1 mm had better survival than patients with CRM 0 mm and CRM 0-1 mm (OS, *P* = 0.005 and *P* < 0.001; DFS, *P* = 0.017 and *P* = 0.001; respectively, log-rank test) (Figs. 4 and 5). However, the difference in OS and DFS between CRM 0 mm and CRM 0-1 mm was not statistically significant within the pN0 group (*P* = 0.476 and *P* = 0.692, respectively, log-rank test). But in the pN1-2 group, patients with CRM 0 mm had worse survival than patients with CRM 0-1 mm and CRM > 1 mm (OS, *P* < 0.001 and *P* < 0.001; DFS, *P* = 0.001 and *P* = 0.005; respectively, log-rank test) (Figs. 4 and 5). And there was no significant difference between CRM 0-1 mm and CRM > 1 mm in OS and DFS within the pN1-2 group (*P* = 0.813 and *P* = 0.194, respectively, log-rank test). And the detailed univariate cox regression analyses data related to OS and DFS in pN0, pN1-2, and pN3 subgroups were shown in Table 5.

**Table 4:** The correlation between CRM status and lymph nodes status and extranodal invasion of lymph nodes metastasis

		n	CRM status			
			0 mm	0-1 mm	> 1 mm	*P* value
pN	N0	405	25(6.2%)	109(26.9%)	271(66.9%)	0.005
	N1	238	11 (4.6%)	77 (32.4%)	150 (63.0%)	
	N2	140	16 (11.4%)	52 (37.1%)	72 (51.4%)	
	N3	55	7 (12.7%)	20 (36.4%)	28 (50.9%)	
Extranodal invasion	No	207	11(5.3%)	66(31.9%)	130(62.8%)	0.056
	Yes	226	23(10.2%)	83(36.7%)	120(53.1%)	

*CRM* Circumferential resection margin

**Table 5:** CRM status and survival adjusted for lymph node status

		N0		N1-2		N3	
		HR (95%CI)	*P* value	HR (95%CI)	*P* value	HR (95%CI)	*P* value
OS							
CAP criteria	R1 vs. R0	1.846(1.067–3.196)	0.029	2.511(1.645–3.835)	< 0.001	1.367(0.576–3.246)	0.478
RCP criteria	R1 vs. R0	1.874(1.366–2.570)	< 0.001	1.122(0.874–1.441)	0.366	1.499(0.830–2.705)	0.179
CRM three-tier criteria	0 mm vs. 0-1 mm	1.236(0.686–2.228)	0.481	2.521(1.590–3.996)	< 0.001	1.112(0.437–2.833)	0.824
	0 mm vs. > 1 mm	2.208(1.258–3.876)	0.006	2.483(1.608–3.835)	< 0.001	1.596(0.642–3.967)	0.314
	0-1 mm vs. > 1 mm	1.801(1.283–2.529)	0.001	0.970(0.741–1.270)	0.825	1.468(0.776–2.777)	0.238
DFS							
CAP criteria	R1 vs. R0	1.615(0.952–2.740)	0.076	1.936(1.271–2.949)	0.002	1.369(0.611–3.071)	0.446
RCP criteria	R1 vs. R0	1.735(1.287–2.339)	< 0.001	0.956(0.751–1.218)	0.716	1.196(0.676–2.115)	0.538
CRM three-tier criteria	0 mm vs. 0-1 mm	1.120(0.634–1.979)	0.695	2.126(1.345–3.359)	0.001	1.266(0.523–3.067)	0.601
	0 mm vs. > 1 mm	1.884(1.097–3.232)	0.022	1.820(1.184–2.800)	0.006	1.440(0.613–3.380)	0.403
	0-1 mm vs. > 1 mm	1.700(1.233–2.345)	0.001	0.845 (0.651–1.097)	0.205	1.123(0.604–2.088)	0.714

*CAP* College of American Pathologists, *CRM* Circumferential resection margin, *RCP* Royal College of Pathologists

## Discussion

The current study evaluated the long-term survival of 838 Chinese ESCC patients to clarify the prognostic value of CRM status. And, the results of our study demonstrated that CRM status was predictive of OS and DFS only in patients with a lower lymph node burden (pN0-2), and the 1 mm-three-tier criteria of CRM status was recommended in clinical practice.

The importance of CRM status in esophageal cancers has been discussed for decades, but remains controversial. Reviewing relevant articles, we found most of the previous reports analyzed different histologic types (mainly adenocarcinoma) as a whole of esophageal cancers [7, 10, 11, 20]. There were only five publications focused specifically on the relationship between CRM status and prognosis of ESCC patients [4–6, 21, 22]. The study by Okada et al. [5] showed that positive CRM was a significant prognostic factor for poor survival, judged by the CAP criteria or the RCP criteria in surgery alone subgroup. However, CRM status, according to the RCP criteria, had no association with survival in patients with neoadjuvant chemotherapy plus surgery [5]. Chao et al. [21] analyzed the relationship between CRM distance and survival in patients with neoadjuvant therapy, and confirmed the 1 mm-three-tier CRM criteria would provide more useful information for risk stratification in cancer recurrence and survival. Whereas, that study only included patients with small number of lymph node metastasis (pN0-1) [21]. Unlike Chao et al.’s results, Park et al. [22] found there were significant relationship between CRM status and loco-regional recurrence in patients with large number of lymph node metastasis (pN2-3), but not pN0-1 [22]. Although the study by Lee et al. [6] showed that patients with positive CRM had worse OS according to both the CAP criteria and the RCP criteria, the 500 μm-three-tier criteria of CRM status provided more detailed prognostic information. Notably, some patients in this study ever received neoadjuvant therapy [6]. Different from the above studies, Yang et al. [4] failed to demonstrate positive CRM, according to either the CAP criteria or the RCP criteria, had significant association with OS in pT3N0M0 ESCC patients, but they found patients with CRM more than 600 μm showed better OS than the ones with CRM less than 600 μm. Based on the previous findings, the impact of lymph node status as confounders of the predictive value of CRM status is uncertain.

Consistent with the previous studies [5, 6, 21], this current study showed CRM status, according to either the CAP criteria, the RCP criteria, or the 1 mm-three-tier criteria, was an independent risk factor affecting patient survival for the entire study. In addition, multivariate analysis showed lymph nodes metastasis was also related to the prognosis of ESCC patients. In order to investigate whether lymph node status would affect the prognostic significance of CRM status, we analyzed the relationship between CRM status and lymph node status. The result showed that there was a significant correlation between CRM status and pN. Subsequently, we performed additional subgroup analyses according to the pN status. Similar to the findings of Yang et al. [4], CRM status, according to either CAP or RCP criteria, was a risk factor affecting the prognosis of patients within the pN0 group, but the survival difference between CRM 0 mm and CRM 0-1 mm was not statistically significant. In the pN1-2 group, patients with CRM 0 mm had worse survival than ones with CRM 0-1 mm and CRM > 1 mm, and patients with CRM 0-1 mm had worse survival than ones with CRM > 1 mm, but the survival difference between patients with CRM 0-1 mm and CRM > 1 mm was not statistically significant. Notably, CRM status, according to either the CAP criteria or the RCP criteria, had no significant association with OS and DFS within the pN3 group. That may indicate that with the number of lymph node metastases increased, the impact of CRM status on prognosis decreased. Especially, the prognosis of patient's prognosis with pN3 is already very poor, the influence of other factors can be masked.

In spite of the present study is the largest number to evaluate the long-term prognostic value of CRM status in patients with pT3 ESCC without neoadjuvant therapy, there are several limitations in our study. First of all, this study failed to accurately collect the recurrence of the tumor and perform relevant statistical analysis. In addition, owing to the retrospective nature of this report, there was wide variation in adjuvant chemotherapy usage and radiation for which we could not account, and most patients received adjuvant therapy because of lymph node metastasis (76.4%). So, the efficacy of adjuvant therapy in positive CRM patients was not well established. Furthermore, given the retrospective nature of the study, and regional lymph nodes were dissected out and sent separately, the CRM status of lymph nodes metastasis could not be accurately assessed. Finally, we did not analyze whether neoadjuvant therapy would affect the prognostic significance of CRM status, because patients with neoadjuvant therapy were excluded in the study.

## Conclusions

CRM status is associated with long-term outcome in ESCC, but only for patients with small number of lymph node metastasis (pN0-2). And we suggest adopting the 1 mm-three-tier criteria of CRM status in clinical practice.

## Data Availability

All data generated or analysed during this study are included in this published article.

## Abbreviations

CRM: Circumferential resection margin
CAP: The College of American Pathologists
RCP: The Royal College of Pathologists
ESCC: Esophageal squamous cell carcinoma
DFS: Disease-free survival
OS: Overall survival
LVI: Lymphovascular invasion
PNI: Perineural invasion
